# Neuropsychology of epilepsy surgery and theory-based practice: an opinion review

**DOI:** 10.1055/s-0043-1770349

**Published:** 2023-10-04

**Authors:** Panayiotis Patrikelis, Lambros Messinis, Vasileios Kimiskidis, Stylianos Gatzonis

**Affiliations:** 1 University of Athens, School of Medicine, Evangelismos Hospital, Epilepsy Surgery Unit, National and Kapodistrian 1st Department of Neurosurgery, Laboratory of Clinical Neuropsychology, Athens, Greece.; 2 Aristotle University of Thessaloniki, School of Psychology, Faculty of Philosophy, Thessaloniki, Greece.; 3 University Hospital of Patras, School of Medicine, Neuropsychology Section, Departments of Neurology and Psychiatry, Patras, Greece.; 4 Aristotle University of Thessaloniki, School of Medicine, Faculty of Health Sciences, 1st Department of Neurology, Thessaloniki, Greece.

**Keywords:** Neuropsychology, Luria-Nebraska Neuropsychological Battery, Epilepsy, Neurosurgery, Information Theory, Neuropsicologia, Bateria Neuropsicológica de Luria-Nebraska, Epilepsia, Neurocirurgia, Teoria da Informação

## Abstract

The present review attempts to discuss how some of the central concepts from the Lurian corpus of theories are relevant to the modern neuropsychology of epilepsy and epilepsy surgery. Through the lenses of the main Lurian concepts (such as the qualitative syndrome analysis), we discuss the barriers to clinical reasoning imposed by quadrant-based views of the brain, or even atheoretical, statistically-based and data-driven approaches. We further advice towards a systemic view inspired by Luria's clinical work and theorizing, given their importance towards our clinical practice, by contrasting it to the modular views when appropriate. Luria provided theory-guided methods of assessment and rehabilitation of higher cortical functions. Although his work did not specifically address epilepsy, his theory and clinical approaches actually apply to the whole neuropathology spectrum and accounting for the whole panorama of neurocognition. This holistic and systemic approach to the brain is consistent with the network approach of the neuroimaging era. As to epilepsy, the logic of cognitive functions organized into complex functional systems, contrary to modular views of the brain, heralds current knowledge of epilepsy as a network disease, as well as the concept of the functional deficit zone.

## Resumo

A presente revisão tenta discutir como alguns dos conceitos centrais do corpus de teorias lurianas são relevantes para a moderna neuropsicologia da epilepsia e cirurgia da epilepsia. Através das lentes dos principais conceitos lurianos (como a análise qualitativa de síndromes), discutimos as barreiras ao raciocínio clínico impostas por visões do cérebro baseadas em quadrantes, ou mesmo abordagens ateóricas, baseadas em estatísticas e orientadas por dados. Aconselhamos ainda uma visão sistêmica inspirada na clínica e na teorização de Luria, dada sua importância para nossa prática clínica, contrastando-a com as visões modulares quando apropriado. Luria forneceu métodos teóricos de avaliação e reabilitação de funções corticais superiores. Embora seu trabalho não abordasse especificamente a epilepsia, sua teoria e abordagens clínicas na verdade se aplicam a todo o espectro da neuropatologia e respondem por todo o panorama da neurocognição. Essa abordagem holística e sistêmica do cérebro é consistente com a abordagem de rede da era da neuroimagem. Quanto à epilepsia, a lógica das funções cognitivas organizadas em sistemas funcionais complexos, ao contrário das visões modulares do cérebro, anuncia o conhecimento atual da epilepsia como uma doença em rede, bem como o conceito de zona de déficit funcional.

## INTRODUCTION

Epilepsy surgery (ES) constitutes an effective alternative treatment option for patients with refractory focal epilepsy. Patients are characterized as pharmacoresistant according to the International League Against Epilepsy (ILAE), definition of drug-resistant epilepsy, 1 that is, all of them were diagnosed with partial epilepsy and continued to have seizures for > 3 years despite the adequate and informative treatment with at least 5 antiepileptic drugs (AEDs).

Advances in surgical techniques have improved seizure control outcomes and the quality of life of patients. 2 3 The presurgical workup is vital for selecting patients and ensuring optimal outcomes, as well as developing a safe and rational surgical strategy. It relies heavily on an interdisciplinary effort encompassing electrophysiological, neuroimaging, neurological, WADA test, neuropsychological and psychiatric evaluation. In particular, the role of neuropsychological assessment in the context of preoperative monitoring of patients with drug-resistant epilepsy could be summarized in two general contexts: on the one hand anatomical localization and hemispherical lateralization of neurocognitive deficits associated with the seizures, and on the other, predictive information about the postoperative outcome of memory and cognitive functions, as well as the effectiveness of surgical treatment in seizure control. More specifically, these two contexts establish the neuropsychologist's specific contribution in four major areas: preoperative and postoperative assessment of neurocognitive function; neurocognitive assessment during the WADA test for the purpose of hemispherical lateralization of speech processes and functional asymmetries of memory; the interpretation of neuropsychological performance of patients undergoing functional magnetic resonance imaging (fMRI) protocols; the intraoperative or perioperative evaluation of cognitive and sensory-motor functions via electrocortical stimulation mapping. 4

Although advanced electrophysiological and neuroimaging methodologies provide high-quality diagnostic data, the information provided by preoperative neuropsychological evaluations may significantly contribute to the characterization of the functional deficit zone, 5 and more specifically cortical areas that are functionally abnormal between seizures, a concept at the heart of focal epilepsy syndromes. This is achieved by documenting and confirming anatomical (topical) information from other measures or even other instances, rejecting, if necessary, some of the earlier localizing scenarios, and functional aspects that otherwise would remain inaccessible to other diagnostic methods (neuroimaging and electrophysiology). 6 7 While in the past neuropsychological evaluation was the gold standard for localizing and lateralizing lesions, with the advent of advanced neuroimaging and electrophysiological methods, contemporary neuropsychological assessment is more likely to deal with other important clinical and rehabilitative issues, that is, neuropsychological differential diagnosis to determine deficit profiles in various neurological (such as dementing conditions) and/or neuropsychiatric (such as psychotic disorders) conditions, informing physicians as to the negative effects of certain types of medications on cognition, establishing performance baselines to detect cognitive decline, planning cognitive rehabilitation protocols on the basis of the neuropsychological pattern of impairment of the patients, providing prognostic information with respect to the vocational, social, and functional outcome after brain injury etc. According to Beaumont, 7 in spite of the considerable advancements, particularly the development of modern medical imaging, clinical neuropsychologists can still make a substantial contribution to the diagnosis and localization of lesions in individual cases.

Despite this shift in the state of clinical affairs, the neuropsychology of ES is a domain in which serious concerns are raised as to the effectiveness of both biomedical technology and mainstream neuropsychology in solving diagnostic dilemmas. A strong critic 8 on the frequent risk of reverse inference in fMRI research outlines the dangers of assuming uniformity across contexts and drawing premature, data-driven, inferences based on activation patterns alone. “… *It is precisely here that the mettle of Luria's contribution to neuropsychology is tested and emerges from the fire as true cultural neuropsychology….”* 9 .

It should not be omitted that besides neuropsychological monitoring, psychiatric assessment is another major although often neglected issue in epilepsy presurgical workup, since active psychopathology may affect postoperative seizure freedom; surgery may lead to the development of de novo psychiatric disorders, and in many instances reverse a preexisting psychiatric condition. This calls for a comprehensive psychiatric assessment of ES candidates pre- and postsurgery to minimize the risk of postsurgical psychiatric morbidities and/or poor quality of life. 10

The literature search conducted in the present paper comprised empirical research studies, reviews, books and chapters along with references included within identified works. The Scopus, PubMed, and Google scholar databases were used as search engines. Only papers in English were considered. The last search was conducted in February 2022. The search terms were the following: *neuropsychology* AND *epilepsy* *surgery* , AND *Luria/Neolurian* / AND *theory* / *model* / *approach* AND *cognitive* / *neuropsychological* AND *memory* / *learning* AND *executive* AND *function/performance* AND *presurgical/preoperative* AND *postsurgical/postoperative* AND *assessment/evaluation/monitoring* AND *temporal lobe epilepsy* / *frontal lobe epilepsy* AND *neuroimaging* . From this search, 7 epilepsy surgery studies, 3 on frontal lobe epilepsy, 11 on temporal lobe epilepsy, 7 on memory and learning, 4 on neuroimaging, 3 on neural networks, and 10 on Luria's work and implications, were fully screened, and related accidents were included.

With the present review, we summarize the central concepts of the Lurian theory (such as the qualitative syndrome analysis), their relevance and implications to the modern neuropsychology of epilepsy and ES underscoring our Lurian-based practice, as well as the barriers to clinical reasoning imposed by quadrant-based views of the brain, or even atheoretical, statistically-based and data-driven approaches. We further advise towards a systemic view inspired by Luria's clinical work and theorizing, given their importance towards our clinical practice, by contrasting it to the modular views when appropriate.

## NEUROPSYCHOLOGICAL TOPICAL DIAGNOSIS IN ES

As already mentioned, one of the main goals of presurgical neuropsychological assessment is to highlight the so-called functional deficit zone, that is, the array of brain areas showing cognitive dysfunction and/or deficits between seizures. This may involve an extensive constellation of regions with either intrahemispheric and/or interhemispheric anatomical distributions. Thus, informing neurosurgeons and neurologists as to the main nodes making up the epileptic network may increase surgical outcome. 4 11

Importantly, this is consistent with the current epistemological trends suggesting a shift from the concept of epileptic focus to that of epileptic network, looking at epilepsy as a neuronal network disease. 12 Moreover, neuropsychological assessment may point to cognitive potentialities or deficiencies that are inconsistent with previous anatomical findings (such as magnetic resonance imaging [MRI] electroencephalogram [EEG]). Such discrepancies are of considerable value since they may show, for instance, an unsuspected atypical representation of language. Hence, integrating neuropsychological findings with data from multiple sources offers a more complete picture for individual patients. 13

Evidence has documented the potential of the preoperative neuropsychological assessment aiding in seizure lateralization when EEG and MRI findings fail to provide clear lateralization data. 14 15 16 17 18 For instance, the Logical Memory subtest of the Wechsler Memory Scale-Revised 19 percent of verbal retention may aid lateralization in temporal lobe epilepsy (TLE) patients for whom MRI findings were insufficient. 20 Another way to obtain lateralization clues is through the use of discrepancy scores on memory measures, such as the Memory Assessment Scales 21 (MAS); Verbal-Visual Memory discrepancy 22 ; and Auditory-Visual Delayed Index difference. 23 Postoperative cognitive outcome prediction in epilepsy is far more developed 24 than the utility of neuropsychological test scores in predicting seizure lateralization and localization in individual patients. 18 Regression formulas have also been proposed to enhance the clinical utility of presurgical neuropsychological data, 18 aiming at identifying which neuropsychological domains contributed with a significant incremental variance to the prediction of seizure lateralization. Although elegant statistical applications may be of aid in isolating cognitive domains accounting for seizure lateralization prediction, they can hardly explain essential qualitative differences; that is, the salience of one or other factor/s apparently leading to the same (epiphenomenon) cognitive domain affection, yet implying the differential contribution of mental component/s breakdown with an altogether different anatomical distribution. Thus, it is difficult, if not impossible, to interpret these findings in the light of a cause and effect relation. For example, correlational data show that a pair of variables may move together (covary) but this is not always equivalent to a causality rapport.

For decades, the neuropsychology of epilepsy has been dominated by modular brain theories (such as the material-specificity theory of memory), which on the one hand have greatly contributed to the understanding of epilepsy-related neurocognitive impairment and its neurobiological determinants, but on the other have posed considerable obstacles to clinical understanding. 25 Instead, Luria looked at higher cortical functions as the result of dynamic and coordinated work of various brain areas, each playing its own particular and unique role as part of a functional system. 26 In fact, he assumed that mental operations are the coproduct of different functional systems, with their components lying on different sides and sites of the brain. Therefore, neuropsychological deficits are no longer regarded in terms of specific brain region malfunction giving rise to symptoms (taxonomic reduction), but rather through a qualitative syndrome analysis or symptom-complex. To Luria, a syndrome is a particular structure resulting from causally related, multi-level symptoms (primary and secondary) collections, enabling the clinician to access the ¨internal geometry¨ of a neuropsychological breakdown and hence advance localization hypothesis. Although a shallow understanding of Luria's methods may regard them as impromptu or even not amenable to quantification, the clinical impact of his theory revolutionized human neuropsychology.

## THE LURIAN MODEL IN EPILEPSY

Luria produced a theory corpus that, although not exclusively focused on epilepsy, appears to apply to the complete range of neuropathology. His holistic and systemic approach of the brain is consistent and to some extent foretold modern network approaches stemming from neuroimaging. Regarding epilepsy, the logic of cognitive functions organized into complex functional networks, contrary to modular views of the brain, seems to herald current knowledge of epilepsy as a network disease, as well as the concept of the functional deficit zone. These contributions seem to be of capital importance for the neuropsychology of ES, since they provide valuable methods and theories to aid in the localization – and lateralization – of cognitive deficits *(see below)* . Consequently, they are of great applicability in the context of the preoperative neuropsychological monitoring of patients-candidates for ES, where neuropsychologists strive towards the anatomical mapping of neuropsychological deficits to aid surgeons. 24

To some authors, conventional neuropsychological measures can hardly differentiate seizures with different focal onseţ with specific associations between neuropsychological deficits and types of epilepsy representing mostly exceptions, 27 28 while the complex interplay between cognitive performance and epilepsy-related variables may obscure the patients' neuropsychological picture, thus further complicating things. For instance, the known negative effects of seizures spreading from temporolimbic to frontal brain regions may explain the secondary-systemic frontal-like dysfunction frequently encountered in TLE patients, and vice versa 29 30 31 (that is, the problem of overlapping deficits). Nevertheless, dichotomous and quadrant-based views continue to pose barriers on the way clinicians interpret cognitive functions and their supposed direct link to specific areas of the brain. This may give rise to arbitrary and oversimplified interpretations of neuropsychological performance based on the distinction between frontal and temporal, or even exclusively hippocampal tasks. 24

This failure in topographic distinction of seizure onset on the basis of test performance-related impairments further emphasizes the need for a wider view of cerebral functional organization extending beyond mere ¨localizationism¨, performance-based, or even preconceived and/or psychometrically-based construct validity assumptions of cognitive measures.

A quick glance at the historical studies of surgical patients suffering from intractable temporolimbic epilepsies 32 may better clarify the issue above. Since then, material-specificity theory of memory was the leading paradigm in the neuropsychology of focal epilepsies, mesial (M)-TLE in particular, pointing on a clear-cut lateralization of cognitive functions and especially of memory. Yet, both clinical practice and neuroimaging research have raised further concerns regarding this theory, 33 proposing, for instance, a dynamic interaction between left and right temporal regions, by selectively engaging on the basis of the task demands at hand. 34 35 36 37 38

The complex inferential reasoning that drives diagnostic hypotheses has progressively been substituted by almost reflexive clinical automatisms, such as those seen when administering standardized cognitive tests and interpreting the data collected, stemming from modular and performance-based neuropsychological approaches. This reflects a rather mechanistic and nontheoretical perspective. Reliance upon diagnostic hypotheses formed on the basis of clinical observation, as well as scientific contextualization of findings and assumptions in the light of a theory are lacking. As Luria 39 states, an important flaw of standardized tests is their reliance on a preconceived classification of “functions”; thus, they are hardly able to reveal the structure of neurocognitive abnormalities resulting from brain lesions. What is more, such measures are not aimed as much at qualitatively analyzing defects as at evaluating the degree of functional impairment of the patient in terms of performance. Hence, they are unsuited for determining the qualitative features – meaning the structure of the disturbance – and are even less suited for analyzing the pathological components responsible for the impairment. Consequently, implementing standardized measures alone for the diagnosis of circumscribed brain lesions or in cases of ES (for identification of the functional deficit zone) are not likely to justify the confidence placed in them.

Interestingly, the current construct of the functional deficit zone, that is, the part of the brain showing dysfunction interictally, as suggested by objective neurological examination, neuropsychological screening, and functional neuroimaging (including fMRIs and FDG (fluorodeoxyglucose) PET (positron emission tomography)-scanning) is consistent with Luria's systemic view. This is of great importance for the neuropsychology of epilepsy and the preoperative neuropsychological monitoring of patients who are candidates for ES, in particular. This is because the neuropsychologist is called to offer the anatomical distribution of cognitive and neurobehavioral dysfunction which manifests itself during the assessment conducted interictally. The areas identified as participating in the expression of neuropsychological impairment/s are thus informative of the functional deficit zone.

## THEORY-DRIVEN NEUROPSYCHOLOGICAL PRAXIS

During the early 1940s, when imaging technologies were not yet available, neuropsychology played a decisive role in helping clinicians localize and/or lateralize cognitive and neurobehavioral impairments caused by brain injuries, thus contributing to clinical decisions in neurology and neurosurgery. The “blind” conditions under which neuropsychological diagnosis was taking place, as imposed by technological gaps, instead of being an obstacle to the development of neuropsychology, often was the very thing that propelled the refinement of clinical acumen and the cultivation of theorizing.

The neuropsychological phenomena that arise spontaneously or are elicited in the context of either the initial clinical history taking or during test performance provide a starting point for theory-based causal attributions regarding the anatomical distribution of deficits. When interpreting neuropsychological findings to provide localizing neuropsychological diagnosis, neuropsychologists often tend to rely on a sort of basic judgment strategy, an “availability heuristic,” that is, group studies that “profile” cognitive dysfunction and/or neuroimaging studies (task performance and regional activations correlations). In contrast, psychometric data should be used to construct a clinical history in the light of a theory model to allow neuropsychologists to reach a scientifically based hypothesis. It is imperative to observe the specific conditions under which a given deficit manifests itself; to conduct qualitative analysis; to theoretically contextualize neuropsychological data; and to coevaluate the data through the “filter” of the patient's clinical-demographic, cultural, and idiosyncratic (personalized data) background to establish cause and effect relationships. 25 The patient's neuropsychological profile should become “clear” not as a result of other investigations, but rather based on a syndrome analysis – a qualitative inquiry aimed at leading directly to the structure of the disturbance – by a disentangling of the/those factor(s) responsible for functional systems breakdown. We suggest that preoperative neuropsychological assessment in its initial phases would be better conducted with the neuropsychologists being blind to other sources of evidence, to avoid biases from predetermined assumptions. 40

## WHY MODULAR APPROACHES MAY NOT SUFFICE?

Memory and its neurobiological foundations could serve to gain insight into Luria's concept of brain function. More specifically, the material-specific theory of anterograde memory was developed and dominated the neuropsychology of epilepsy, 32 claiming that whenever epilepsy onset originated from the dominant (usually left) temporal lobe, then verbal learning and memory would be adversely affected. Instead, in cases of right nondominant temporal lobe seizure onset, learning and memory for nonverbal material (such as designs or faces) would be affected, although evidence supporting this is weaker than that regarding the left temporal lobe and verbal memory. 41 Furthermore, other cognitive abilities were presumed to remain relatively intact since seizure onset and focal epileptiform abnormalities were thought to exclusively concern the temporolimbic territories supporting encoding new information into memory. The material-specificity memory model was the main indicator of whether the contralateral nonepileptic temporal lobe was functionally enough to sustain memory post-operatively. 42

Consequently, illusory assumptions of a compartmentalization of cognition, that is, verbal versus nonverbal memory and mnemonic versus nonmnemonic functions) led scholars to think for the former a clear-cut left to right temporal lobe functional distinction, while for the latter a frontal to extra frontal dichotomy. Such quadrant-base views, although on one hand being initially an aid to understand brain function, progressively limited clinical thought, often leading to oversimplified and fragmentary views of higher cortical functions. The implications for the presurgical neuropsychological assessment (targeting localization and lateralization of cognitive deficits) were translated into a sort of rigid clinical automatisms dictating a “left to right” and “anterior to posterior” diagnosis of cognitive dysfunction, thus restricting finer and patient-tailored qualitative views. The aforementioned assumptions constitute the main reason for the pressing need of more systematic views, like syndrome analysis stemming from Lurian theory. It means that the anatomical basis of neuropsychological deficits is no longer identified within specific brain sites of the dominant or nondominant hemisphere responsible for the symptom, but rather through a qualitative neuropsychological analysis of the syndrome or symptom-complex. 26

Syndrome analysis is a process of analytic comparison of neuropsychological evidence accessed through various tests and the determination of general signs among them, hopefully defining a unified syndrome. To Luria, a syndrome is conceived as a structure emerged by constellations of causally related, multi-level symptoms (primary and secondary); thus, understanding the different nature of the latter is of crucial importance in determining the source of a neurological breakdown and also in advancing the localization hypotheses. 39

## DIFFERENT DIAGNOSTIC APPROACHES IN NEUROPSYCHOLOGY

Historically, neuropsychological methods of investigation have been linked to three different scientific traditions (North American, Russian – former Soviet Union, and British) that have influenced clinical neuropsychological praxis. While each of them places the emphasis on different constructs, they are hardly independent from each other. 7

The North American approach makes use of selected tests because of their assumed relation to some element of a scheme of psychological abilities. It has been originally developed to assist psychological assessment of individual differences, a topic that received lots of interest by American psychologists. A strong point of such an approach, adopting test batteries, is a comprehensive coverage of functions, allowing the use of scores emerging from different test results. Instead, a weak point links to the extreme length of assessment procedures, their impracticality, as well as the fact of being derived from models of normal mental function, and thus probably not suitable for clinical settings.

On the contrary, the Russian approach to neuropsychology has mainly adopted a single case study methodology stemming from behavioral neurology and consistent with the Lurian theory of brain functions organized in terms of functional systems distributed to particular brain regions. It is based on models of abnormal function, and aiming at the assessment of the functional systems (searching the exact factor/s breakdown leading to the particular disorder) and therefore more suitable for clinical settings. Moreover, tests selection is case sensitive contrasting with the massive use of neuropsychological batteries often administered in an acritical fashion. Tests were rather informal, and overall unstandardized, making little if no use of standardized procedures or normative data (difficult to quantify subtle changes but more sensitive to qualitative aspects of behavior), while successful diagnosis heavily relied on the level of expertise of the clinician.

British neuropsychology stands between these two approaches, investigating individual cases with selected standardized measures. The main advantage of this approach is its special focus on individual patients and the parallel use of statistical analysis, thus being able to profile the disability of individual patients. However, the investigative process may be fragmentary and unsystematic, often placing huge reliance on tests procedures that happen to be available and, probably, therefore insensitive and/or inadequate. 8

It is important to note that all approaches rely to some extent on the clinical expertise and acumen of the neuropsychologist. During the past two decades, the British approach was the dominant international style, mostly because of the growing influence of cognitive neuropsychology.

Diagnostic approaches in neuropsychology are likely to vary in accordance to their theory framework of reference. On our opinion there is a general state of confusion regarding the very meaning and implications of neuropsychological theories and the methodological foundations of neuropsychology. Many people believe that by making use of normative data, validated methods of investigation or evidence-based knowledge may suffice or even remedy for the lack of theory. To this inconvenience may contribute the current state of affairs in clinical neuropsychology being dominated by statistically-based, data-guided or even atheoretical views. Instead, Luria proposed a conceptually-driven clinical praxis in neuropsychology. He was inclined to strongly reject an approach in which “auxiliary aids become the central method and in which their role as servant to clinical thought is reversed so that clinical reasoning follows instrumental data as a slave follows its master” 43 . Evidently, Luria's perspective has been strongly impacted by eminent physiological theories (such as the Pavlovian law of power), his Marxist views and the implementation of historical materialism to the study of the brain to determine cause and effect relations.

The Lurian approach has been widely criticized since the lack of a direct evaluation of the tests and of controlled scoring, with the latter mainly based upon the level of expertise of the clinician rather than on normative data. Standardized measures have proven to be more valid and reliable in cases of focal, well-defined brain insults, but with limited capacity when assessing patients suffering ill-defined brain impairments (as in many epilepsy patients) and severe or diffuse neurobehavioral disorders. 44 However, when examining neurocognitive functions using standard measures on two different patients who have been assigned the same degree of severity, they may manifest qualitatively different neuropsychological deficits. In effect, well-known cognitive constructs may become blurred due to the weak correlation between paradigms with multiple cognitive factors, and performance in different cognitive tasks. 45 On the contrary, Luria poses the emphasis on the process (not on test achievement) allowing for error analysis and shared breakdowns (factors) across tasks, to enable clinicians to identify the impaired network. Therefore, it proves more apt for patients with diffuse neuropsychological impairments resulting from network disconnection, as in epilepsy, given the disconnecting interfering effects of seizures on functional networks. Various attempts have been undertaken to standardize Luria's tests, with the work of Glozman 46 being the most representative, while many neuropsychologists have started to consider rather flexible assessment protocols by choosing both quantitative and qualitative tests from different batteries, targeting (according to Luria) a synthetic evaluation of cognitive functions and making it possible to dissect them into several functional domains.

The dichotomy between quantitative and qualitative neuropsychology is an illusory one, since the qualitative approach is nothing more than an approach based on productive, positive symptomatology, which is susceptible to quantification. A necessary compromise of psychometric tradition with the qualitative approach is expected by modern neuropsychological procedures offering both symptom elicitation and quantification. 47

## WHAT ABOUT EVIDENCE-BASED NEUROPSYCHOLOGY?

According to Chelune, 40 evidence-based clinical neuropsychological practice (EBCNP) refers to methods for enhancing interactions between research and practice (clinical outcomes research), that is, “… *the scientific method applied at the level of the individual hypothesis formation, literature review, study design and data collection, analysis, and conclusion* …”. Thus, it is a mistake confounding EBCNP with the adoption of a particular neuropsychological theory, whatever it is. Evidence-based clinical neuropsychological practice refers to a method rather than a theory framework. When it comes to epilepsy, evidence-based neuropsychological knowledge (largely based on group studies) indicates, for instance, a lack or even absence of differentiation in terms of cognitive performance between focal epilepsy syndromes (such as FLE versus TLE), despite some significant differences found in individual subtests, 48 49 leaving a knowledge gap in preoperative neuropsychological diagnosis of the functional deficit zone. We believe that a theory-guided approach (such as that proposed by Luria for syndrome analysis) could bridge this gap. Consequently, as we did elsewhere, 16 we strongly propose that contextualized interpretation, based on theories, should also be adopted by neuropsychologists working in the domain of ES.

## RESEARCH EVIDENCE LEGALIZING LURIA

In recent times, the functional systems theory of cerebral organization has been in part refined and further elaborated from neolurians who continued this school of thought in neuropsychology, 50 51 52 as well as by others who inadvertently arrived at the same conclusions regarding brain function. 53 Goldberg et al. 52 proposed a Lurian-based theoretical extension to account for hemispheric specialization in terms of novelty/familiarity distinctions as opposed to material specificity theory, that is, spatial versus. linguistic or global versus local processing.

Goldberg's gradiental theory constitutes a rejection of the modular view proposing a distributed-emergent principle, accounting for cortical functional organization. 50 51 53 He proposes a spectral anatomical distribution of heteromodal association areas, called gradients, housing functionally similar cognitive processes in anatomical proximity within association cortices. Another theory of cortical representation elaborating on Luria's functional systems theory was formulated by Fuster. 53 In his view, reiterant units called *cognits* comprise cognitive functions being intended as information exchange within and between cognits. The crucial element here is that different cognits (neural networks) have an identifiable cortical distribution, but cognitive functions do not, since the later are mediated both by shared and/or similar circuits.

There are also studies replicating early theoretical notions as those put forward by Elkhonon Goldberg (one of Luria's students), who hypothesized differences in hemispheric specialization arising as a function of the practical acquisition and use of descriptive systems (such as including language and other symbolic systems). 50 Accordingly, early bilingualism not only induces plastic changes within language networks, but also in those mediating executive functions (for review, see 54 ). Similar long-term effects inducing neuroplastic changes have been reported in cases of chronic practice of focused tasks, such as musical performance 55 56 and mental calculations on an abacus. 57 It was also put forward that physical environment seems to determine one's initial preference and its later development for adopting either ventral stream (system of what) or dorsal stream (system of where) processing styles when conducting visual inspection. 58 59 60 61 62

Aversi-Ferreira 63 reviewed Luria's studies on the neuropsychology of the temporal lobes and compared these with more recent data. The authors showed that Luria's theory constitutes the basis for neuropsychological studies today, while new imaging data on the temporal lobe in relation to epilepsy and hippocampus analysis are consistent with Luria's views. 26

Current neuroimaging research points to the failure of strict modularity assumptions in more complex integrative tasks, frequently employing analyses of functional connectivity presenting huge analogies and actually formalizing Lurian concepts. 64 A genome-wide focus on neuropsychological phenotypes 65 essentially offers a modern translation of Luria's cultural neuropsychology.

The present paper, through the lenses of the Lurian approach, points to the necessity for the neuropsychology of ES to bypass the quadrant-based views of brain function, in favor of systemic ones like the *syndrome analysis* . Luria's idiographic neuropsychological approach, through thorough and insightful observations and theory-driven methods, may be of considerable aid in the context of the preoperative work-up of ES candidates, particularly when lateralization and localization of seizures is required. We encourage people working in the area of neuropsychology of epilepsy to become familiar with theories of brain function and implement this knowledge to support their clinical decisions.

In conclusion, a neuropsychological approach to epilepsy, consistent with the Lurian view of higher cortical functions organized into functional systems, may deepen the understanding of neurocognitive impairments in patients with epilepsy and surpass the limits imposed by quadrant-based approaches to the brain. 41 Luria's theoretical constructs heralded modern era neuropsychology moving from lesion-studies to a more network-based view of the brain, paralleling the shift in epileptology from the concept of epileptic focus to that of epileptic network. The major advantages of the Lurian approach are its well-structured and well-defined integrative cognitive components and its ability to provide valuable information concerning their interactions. 66 Integration between quantitative and qualitative assessment methods still remains an open issue in clinical neuropsychology, the solution of which would further enhance the potential of Luria's neuropsychological examination paradigms.

Manifold evidence such as hemispheric specialization, neocortical functional organization, functional reorganization and neuroplasticity, visual perception processing styles, bilingualism and neuroplasticity, focused activities and long-lasting neuroplastic changes research and cognitive phenotypes, and temporal lobes neuropsychological diagnostics, legalize current applications of Lurian theory penetrating the whole neuropathology spectrum and accounting for the whole panorama of neurocognition.
